# Reactive Oxygen Species Are Required for 5-HT-Induced Transactivation of Neuronal Platelet-Derived Growth Factor and TrkB Receptors, but Not for ERK1/2 Activation

**DOI:** 10.1371/journal.pone.0077027

**Published:** 2013-09-27

**Authors:** Jeff S. Kruk, Maryam S. Vasefi, John J. Heikkila, Michael A. Beazely

**Affiliations:** 1 Department of Biology, University of Waterloo, Waterloo, Ontario, Canada; 2 School of Pharmacy, University of Waterloo, Waterloo, Ontario, Canada; University of Nebraska Medical Center, United States of America

## Abstract

High concentrations of reactive oxygen species (ROS) induce cellular damage, however at lower concentrations ROS act as intracellular second messengers. In this study, we demonstrate that serotonin (5-HT) transactivates the platelet-derived growth factor (PDGF) type β receptor as well as the TrkB receptor in neuronal cultures and SH-SY5Y cells, and that the transactivation of both receptors is ROS-dependent. Exogenous application of H_2_O_2_ induced the phosphorylation of these receptors in a dose-dependent fashion, similar to that observed with 5-HT. However the same concentrations of H_2_O_2_ failed to increase ERK1/2 phosphorylation. Yet, the NADPH oxidase inhibitors diphenyleneiodonium chloride and apocynin blocked both 5-HT-induced PDGFβ receptor phosphorylation and ERK1/2 phosphorylation. The increases in PDGFβ receptor and ERK1/2 phosphorylation were also dependent on protein kinase C activity, likely acting upstream of NADPH oxidase. Additionally, although the ROS scavenger *N*-acetyl-l-cysteine abrogated 5-HT-induced PDGFβ and TrkB receptor transactivation, it was unable to prevent 5-HT-induced ERK1/2 phosphorylation. Thus, the divergence point for 5-HT-induced receptor tyrosine kinase (RTK) transactivation and ERK1/2 phosphorylation occurs at the level of NADPH oxidase in this system. The ability of 5-HT to induce the production of ROS resulting in transactivation of both PDGFβ and TrkB receptors may suggest that instead of a single GPCR to single RTK pathway, a less selective, more global RTK response to GPCR activation is occurring.

## Introduction

Serotonin (5-HT) is a tryptophan-derived signaling molecule best known for its role as a neurotransmitter [1]. In the central nervous system (CNS), it is involved with a variety of functions including circadian rhythm, mood, memory, and cognition [2–4]. The role of 5-HT in CNS pathology is of particular interest given the fact that there are several examples of clinically used drugs that target the 5-HT system for the treatment of depression, schizophrenia, and other CNS diseases [2,5]. 5-HT binds and activates seven different receptor subtypes including six G protein-coupled receptors (GPCRs) comprising subtypes 1-2 and 4-7, and 5-HT_3,_ a ligand-gated ion channel [6].

The platelet-derived growth factor type β (PDGFβ) receptor is an important receptor tyrosine kinase (RTK) for the development of the CNS [7,8]. Four isoforms of PDGF ligands exist as hetero- or homodimers that bind to the extracellular ligand-binding domains of the receptor [9]. Ligand binding results in the dimerization and activation of the receptor, which triggers intracellular kinase domain-mediated trans-autophosphorylation of several tyrosine residues [7]. Multiple intracellular signaling pathways are initiated that result primarily in the promotion of cell growth [7], however the roles of PDGF signaling in the developed CNS have not been fully elucidated. In addition to direct ligand activation, RTKs like the PDGFβ receptor can be activated in a ligand-independent manner through a process known as transactivation. Transactivation of RTKs is initiated by the activation of GPCRs by ligands such as 5-HT [10,11], dopamine [12], angiotensin II [13], sphingosine-1-phosphate [14], lysophosphatidic acid [15], and leukotrienes [16]. The magnitude of activation of the PDGFβ receptor during transactivation (as measured by tyrosine phosphorylation) is typically much less than ligand-induced activation [10]. This may explain why ligand-induced activation results in rapid down-regulation of RTKs such as the PDGFβ receptor [9], whereas down-regulation of transactivated PDGFβ receptors has not been observed [10,17].

The receptor tyrosine kinase TrkB is activated by brain-derived neurotrophic factor (BDNF) and neurotrophin-4 as well as neurotrophin-3 [18]. TrkB receptors can also be transactivated by adenosine A_2A_ receptors and many of the proteins involved in that pathway are similar to those required for 5-HT-induced transactivation of the PDGFβ receptor [10,19,20]. One of the main components of the neurotrophic factor hypothesis of depression suggests that a reduction of neurotrophic factor signaling, including BDNF, contributes to synaptic dysregulation and neuronal dysfunction [18]. Conversely, the older monoamine hypothesis of depression posits that imbalances in serotonergic systems contribute to depression, with serotonin being the key dysregulated neurotransmitter [21]. A clearer understanding of the signaling relationships between the serotonergic, neurotrophic factor, and neuronal growth factor systems may provide insights into how these two hypotheses of depression could be reconciled.

We have previously shown that 5-HT-induced PDGFβ receptor transactivation involves Gα_i_-coupled 5-HT receptors including 5-HT_1A_ receptors in SH-SY5Y cells [10]. This pathway was sensitive to PLC inhibition and intracellular, but not extracellular, calcium chelation [10]. Previous studies have suggested that ERK1/2 is phosphorylated as a downstream consequence of RTK transactivation [12,22,23]. Interestingly, although we demonstrated ERK1/2 phosphorylation was indeed observed after 5-HT treatment, it was PDGFβ receptor-independent [10]. The current study investigates the role of reactive oxygen species (ROS) in the transactivation of RTKs in neurons. We demonstrate that PDGFβ and TrkB receptors can be transactivated by 5-HT in neuronal cultures and that the transactivation of these RTKs requires ROS and NADPH oxidase activity, however 5-HT-induced ERK1/2 activation is not ROS-dependent.

## Materials and Methods

### Reagents and Antibodies

5-HT (5-hydroxytryptamine hydrochloride), *N*-acetyl-l-cysteine (l-α-acetamido-β-mercaptopropionic acid), diphenyleneiodonium chloride, AG 1296 (6,7- dimethoxy- 2- phenylquinoxaline), Go 6983 (3-[1-[3-(dimethylamino)propyl]-5-methoxy-1H-indol-3-yl]-4-(1H-indol-3-yl)-1H-pyrrole-2,5-dione) and pertussis toxin were purchased from Cedarlane (Burlington, ON). Apocynin (4'-hydroxy-3'-methoxyacetophenone) was purchased from Santa Cruz (Santa Cruz, CA). Antibodies against β-actin, TrkB, PDGFβ receptor, and phospho-PDGFβ receptor Y1021 were also purchased from Santa Cruz. Antibodies against phospho-TrkB Y816, ERK1/2 and phospho-ERK1/2 were purchased from Cedarlane.

### SH-SY5Y cultures

SH-SY5Y cells were obtained as a generous gift from Dr. Shilpa Buch, University of Nebraska. Cultures were grown in complete growth media consisting of DMEM and Ham’s F12 in a 1:1 ratio, 10% fetal bovine serum (Sigma, Oakville, ON), and penicillin/streptomycin. Cultures were maintained in a humidified atmosphere of 95% air and 5% CO_2_ at a temperature of 37°C, with media changes every 3-5 days. For experimentation, cells were plated without antibiotics, and prior to drug treatments, cells were serum starved for 24 h.

### Primary mouse cortical neuron cultures

CD-1 mouse embryos (Harlan, Indianapolis, IN) were removed at E17 to E19 and transferred to chilled dissection media (33 mM glucose, 58 mM sucrose, 30 mM HEPES, 5.4 mM KCl, 0.44 mM KH_2_PO_4_, 137 mM NaCl, 0.34 mM Na_2_HPO_4_, 4.2 mM NaHCO_3_, 0.03 mM phenol red, pH 7.4, 320-335 mOsm/kg). The brains were removed, and the cortex was dissected and trypsinized with 0.25% trypsin for 20 min at 37°C. Cells were then strained and plated on poly-d-lysine-coated culture dishes and grown at 37°C in a humidified atmosphere of 95% air and 5% CO_2_. Cells were plated with plating media (DMEM, supplemented with 10% fetal bovine serum, 10% horse serum) for the first 2-4 h until cells attached. Media were then replaced with feeding media consisting of Neurobasal medium and B-27 supplement (Life Technologies, Burlington, ON) without serum, and half of the media volume per well was changed twice per week. Drug treatments were performed 7-8 days after plating the cells. To prevent the overgrowth of non-neuronal cells, a mitotic inhibitor (81 µM 5-fluoro-2’-deoxyuridine and 200 µM uridine added to media) was added for 24 h once cells reached confluency. All animal experiments were performed in strict accordance with the guidelines and policies on the Use of Animals at the University of Waterloo, and all efforts were made to minimize discomfort. The protocol was approved by the Waterloo Office of Research Ethics Animal Care Committee (Animal Utilization Project Proposal 09-17, 2009-2013).

### Western blotting and data analysis

Following drug treatments, cells were washed once with ice-cold PBS. Chilled lysis buffer (20 mM Tris-HCl at pH 7.5; 150 mM NaCl; 1 mM EDTA; 1 mM EGTA; 30 mM sodium pyrophosphate; 1 mM β-glycerophosphate; 1 mM sodium orthovanadate; 1% NP-40; supplemented with Halt Protease and Phosphatase Inhibitor (Thermo, Fisher, Pittsburgh, PA) prior to use) was added and lysates were homogenized and centrifuged at 13,000 x g for 20 min at 4°C. Supernatant protein concentration was determined using the BCA protein assay (Thermo, Fisher) and samples were normalized. Loading buffer (240 mM Tris-HCl at pH 6.8, 6% w/v SDS, 30% v/v glycerol, 0.02% w/v bromophenol blue, 50 mM DTT, 5% v/v β-mercaptoethanol) was added to samples, which were then heated for 15 min at 75°C. SDS-PAGE was used to separate proteins followed by transfer of proteins to nitrocellulose or PVDF membranes. 5% non-fat milk in Tris-buffered saline plus 0.1% Tween (TBS-T) was used to block membranes for 1 h at room temperature or overnight at 4°C. Membranes were then incubated with primary antibody for 1 h at room temperature or overnight at 4°C. Membranes were washed three times with TBS-T, and then incubated with secondary antibody conjugated to horse radish peroxidase (HRP) for 1 h at room temperature. Membranes were washed three additional times with TBS-T. Proteins were visualized with western chemiluminescent substrate (Millipore, Billerica, MA) on a Kodak 4000MM Pro Imaging Station. Kodak Molecular Imaging software was used for densitometric analyses of images and data statistics were evaluated with GraphPad Prism software with statistical significance set at p < 0.05. After imaging, membranes were stripped and re-probed with other antibodies.

### MTT cell viability assay

SH-SY5Y cells were seeded at equal concentrations and grown to 90% confluency, followed by overnight serum starvation. After H_2_O_2_ treatments, media was changed to serum-free, phenol red-free DMEM/F12 and cultures were returned to the cell culture incubator for 24-48 h to allow mitochondrial enzyme deactivation in non-viable cells. MTT reagent (thiazolyl blue tetrazolium bromide: 3-(4,5-dimethyl-2-thiazolyl)-2,5-diphenyl-2H-tetrazolium bromide; Sigma) was then added to cell culture media, and plates were returned to the cell culture incubator for 2-4 h for the reaction to occur. Cells were then lysed and resulting crystals dissolved in solubilization buffer (0.1 M HCl, 10% Triton X-100 in propan-2-ol) on a gyratory plate shaker. Plates were read at 570 nm absorbance and background absorbance at 690 nm was subtracted from these values.

## Results

### H_2_O_2_ increases PDGFβ receptor phosphorylation

**Figure 1:**
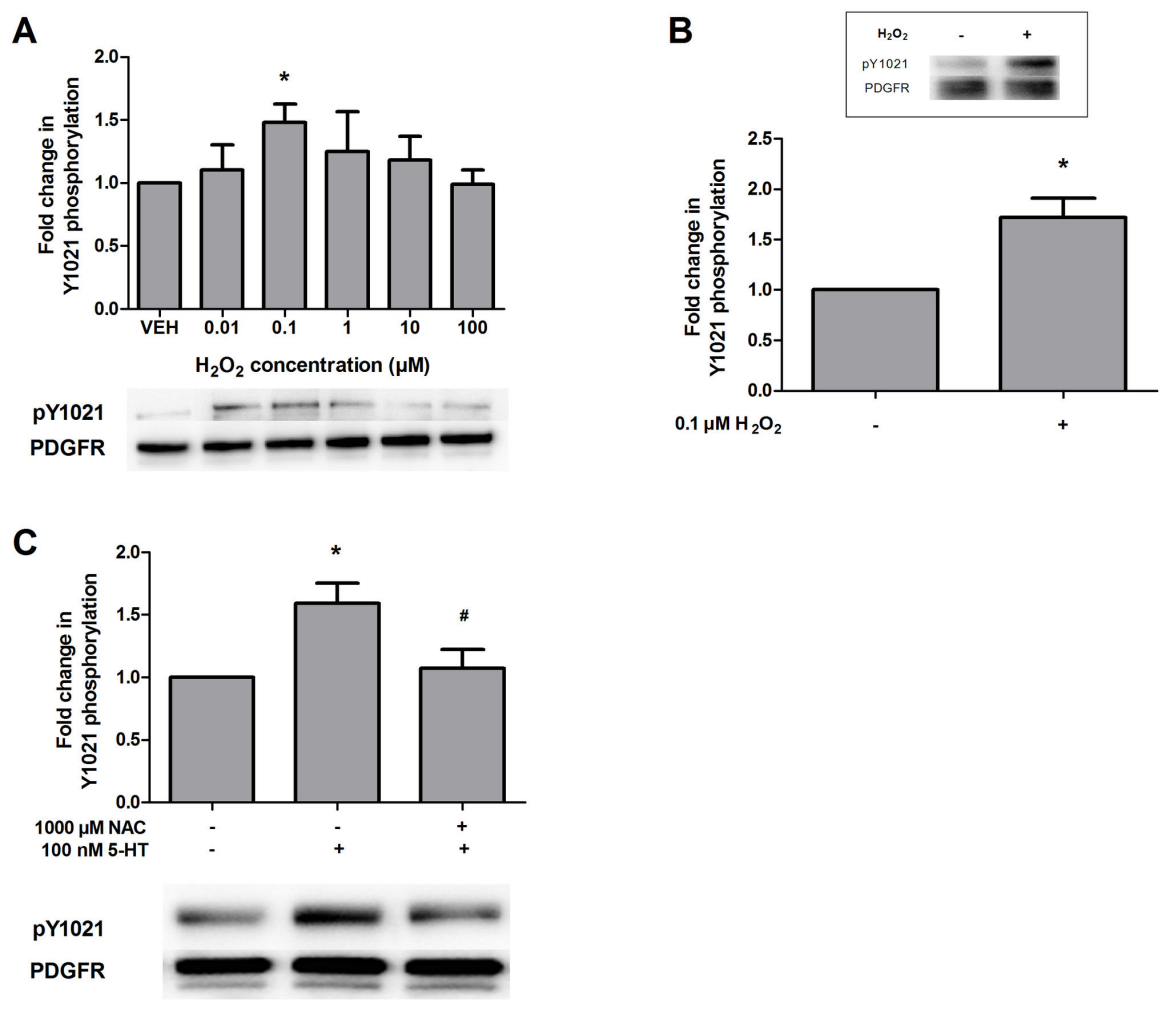
H_2_O_2_ increases PDGFβ receptor phosphorylation in SH-SY5Y cells and primary neuron cultures. (A) SH-SY5Y cells were treated with vehicle (VEH) or 0.01 to 100 µM H_2_O_2_ for 5 min. Following drug treatments, cell lysates were evaluated by Western blot analysis as described in Materials and Methods. Data were normalized to total PDGFRβ protein expression and are expressed as the fold change (average ± S.E.M.) in phospho-1021 immunoreactivity compared to vehicle-treated cells. Representative blots for phospho-PDGFRβ 1021 (pY1021) and PDGFRβ at 180 kDa are shown. (B) Primary mouse cortical neuron cultures were treated with 0.1 µM H_2_O_2_ for 5 min. Lysates were evaluated for phospho-Y1021 as described in “A”. (C) SH-SY5Y cell cultures were pretreated with vehicle or 1000 µM of the ROS scavenger *N*-acetyl-l-cysteine (NAC) for 45 min followed by treatment with vehicle or 100 nM 5-HT for 5 min. (Data are representative of 4-6 independent experiments. * = p < 0.05 compared to vehicle-treated cells; # = p < 0.05 compared to 5-HT-treated cells, one-way ANOVA, Tukey post-test, or Student’s t-test).

**Figure 2:**
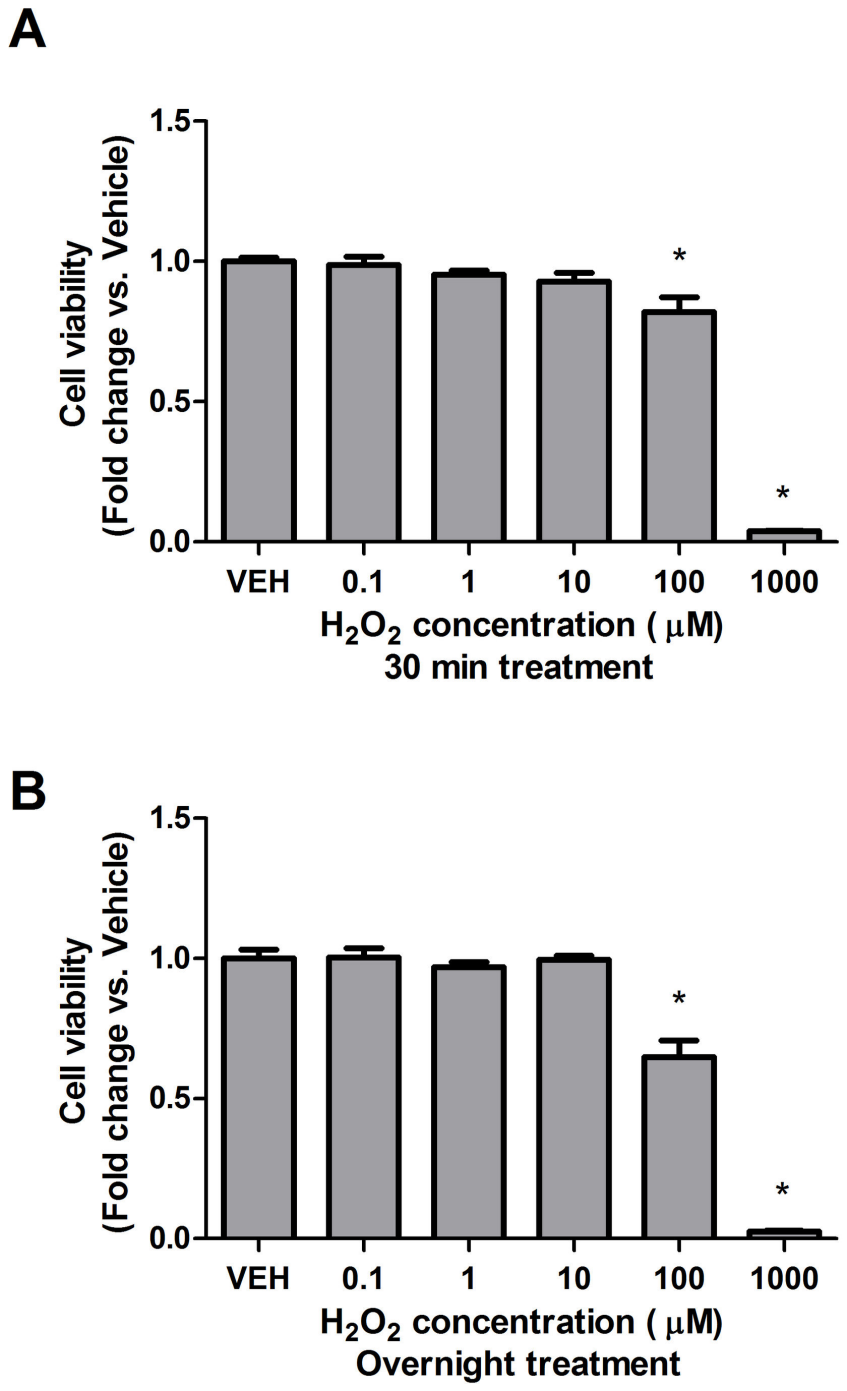
H_2_O_2_ concentrations sufficient for inducing PDGFβ receptor phosphorylation do not result in cell death. SH-SY5Y cells were treated with 0, 0.1, 1, 10, 100, or 1000 µM H_2_O_2_ for (A) 30 min, or (B) overnight. Following treatment with MTT reagents and lysis, cell viability was measured and compared to control (VEH) values. (Data are representative of 4 independent experiments. * = p < 0.05 compared to vehicle-treated cells, one-way ANOVA, Tukey post-test).

We have previously shown that 5-HT increases PDGFβ receptor phosphorylation in both the neuroblastoma-derived SH-SY5Y cell line and primary mouse cortical neuron cultures [10]. Based on transactivation pathways described in other cell types [11,24], we postulated that reactive oxygen species (ROS) are involved in the 5-HT-induced transactivation of neuronal PDGFβ receptors. Since H_2_O_2_ can cross the cell membrane [25,26], we analyzed a dose response of exogenously applied H_2_O_2_ to SH-SY5Y cells for 5 min and observed peak tyrosine 1021 phosphorylation of PDGFβ receptor at a concentration of 0.1 µM (Figure 1A). This concentration was also sufficient to cause transactivation of PDGFβ receptors in primary mouse cortical neuron cultures (Figure 1B). To determine if 5-HT-induced transactivation of PDGFβ receptors involved the generation of endogenous ROS, we pretreated the cells with the ROS scavenger, *N*-acetyl-l-cysteine, followed by 100 nM 5-HT for 5 min (Figure 1C) (we previously determined that this concentration and incubation time of 5-HT resulted in maximal PDGFβ receptor transactivation in these cells [10]). *N*-acetyl-l-cysteine (1000 µM) was able to abrogate PDGFβ receptor phosphorylation, suggesting that ROS are indeed involved in 5-HT-induced PDGFβ receptors transactivation. Because H_2_O_2_ can cause cell damage and death at high concentrations, we verified that the low concentrations of H_2_O_2_ used here (particularly, the concentration of 0.1 µM that induced PDGFβ receptor phosphorylation) were not adversely affecting cell viability. As determined by the MTT cell viability assay, we found that the cells were unaffected by H_2_O_2_ treatment after 30 min (Figure 2A) or overnight treatment (Figure 2B) at concentrations less than 100 µM.

### The role of NADPH oxidase in PDGFβ receptor transactivation

**Figure 3:**
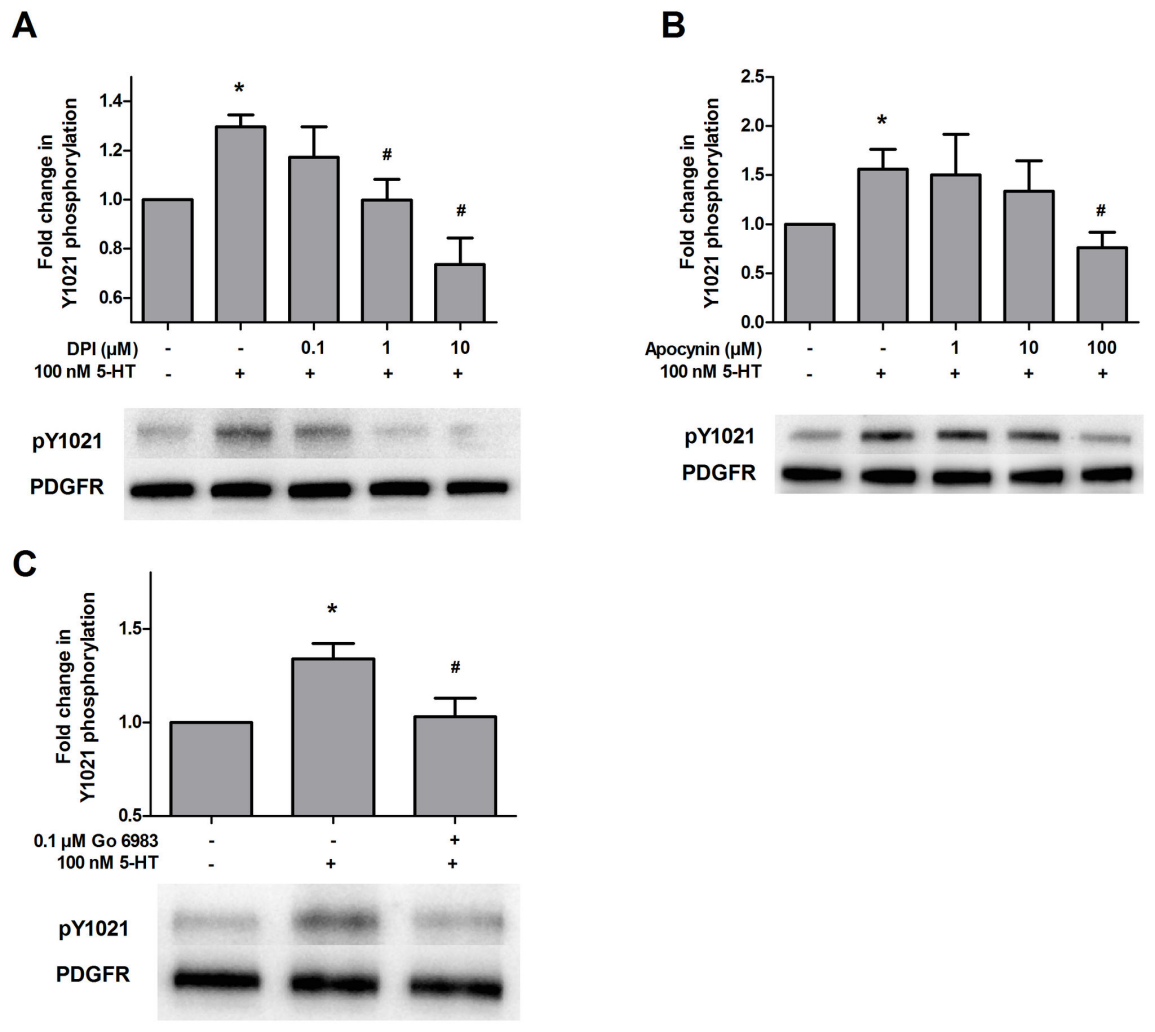
5-HT-induced PDGFβ receptor transactivation requires PKC and NADPH oxidase. (A) SH-SY5Y cell cultures were pretreated with vehicle or 0.1, 1 or 10 µM of the NADPH oxidase inhibitor diphenyleneiodonium chloride (DPI) for 45 min followed by treatment with vehicle or 100 nM 5-HT for 5 min. Following drug treatments, cell lysates were evaluated by immunoblot analysis as described in Materials and Methods. Data were normalized to total PDGFRβ protein expression and are expressed as the fold change (average ± S.E.M.) in phospho-1021 immunoreactivity compared to vehicle-treated cells. Representative blots for phospho-PDGFRβ 1021 (pY1021) and PDGFRβ at 180 kDa are shown. (B) Cell cultures were pretreated with vehicle or 1, 10 or 100 µM of the NADPH oxidase inhibitor apocynin for 45 min followed by treatment with vehicle or 100 nM 5-HT for 5 min, and results were analyzed for phospho-Y1021 as described in “A”. (C) Cultures were pretreated with vehicle or 0.1 µM of the PKC inhibitor Go 6983 for 45 min followed by treatment with vehicle or 100 nM 5-HT for 5 min, and results were analyzed for phospho-Y1021 as described in “A”. (Data are representative of 3-5 independent experiments. * = p < 0.05 compared to vehicle-treated cells; # = p < 0.05 compared to 5-HT-treated cells, one-way ANOVA, Tukey post-test).

To investigate the source of ROS, we considered NADPH oxidase since it has been previously implicated in growth factor receptor transactivation in fibroblasts and keratinocytes [27,28]. Treatment with the NADPH oxidase inhibitors, diphenyleneiodonium chloride (1 µM and 10 µM) or apocynin (100 µM) blocked PDGFβ receptor transactivation by 5-HT (Figure 3A and 3B). In addition, NADPH oxidase components have been shown to be activated by protein kinase C (PKC) [29], either directly or via Rap1A and Rac1/2 [30,31]. We have previously demonstrated that the PDGFβ receptor transactivation pathway initiated by 5-HT involves phospholipase C (PLC) activity and intracellular calcium [10], both of which could lead to the activation of calcium-dependent PKC isoforms [32]. When cells were pretreated with the PKC inhibitor Go 6983 (0.1 µM), 5-HT failed to transactivate the PDGFβ receptor (Figure 3C). These findings, coupled with our previous results, suggest that 5-HT treatment leads to the activation of PKC via PLC and intracellular calcium release, the assembly and activation of NADPH oxidase complex, the production of ROS, and ultimately the phosphorylation of PDGFβ receptor.

### 5-HT also transactivates TrkB receptors

**Figure 4:**
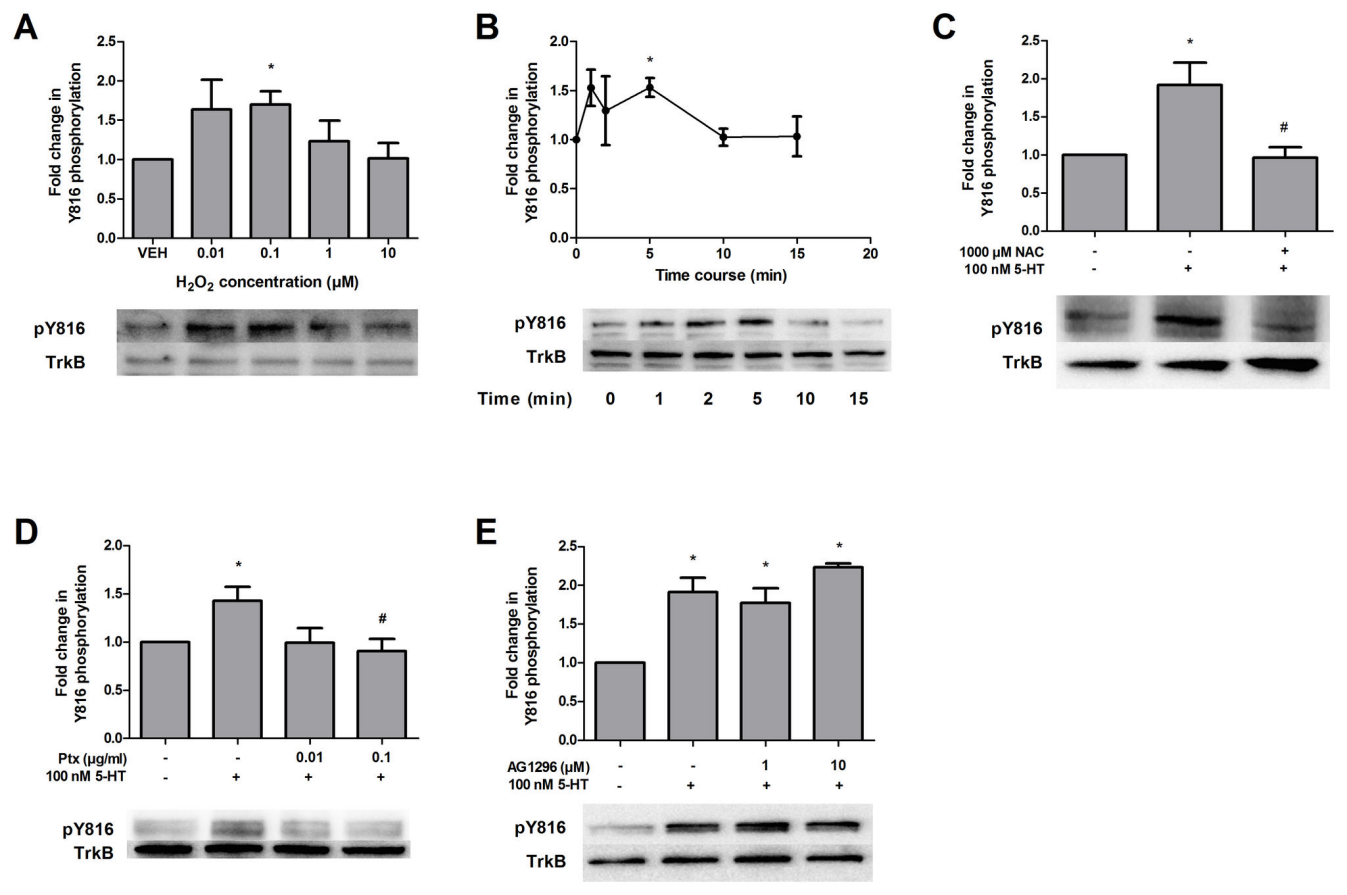
5-HT can transactivate TrkB receptors via ROS. (A) SH-SY5Y cells were treated with vehicle (VEH) or 0.01 to 10 µM H_2_O_2_ for 5 min. Following drug treatments, cell lysates were evaluated by Western blot analysis as described in Materials and Methods. Data were normalized to total TrkB protein expression and are expressed as the fold change (average ± S.E.M.) in TrkB phospho-816 immunoreactivity compared to vehicle-treated cells. Representative blots for phospho-TrkB Y816 (pY816) and TrkB at 145 kDa are shown. (B) Cell cultures were incubated with 0.1 µM 5-HT for 0, 1, 2, 5, 10, or 15 min, and fold change in TrkB Y816 phosphorylation was measured with respect to vehicle. (C) Cultures were pretreated with vehicle or 1000 µM of the ROS scavenger *N*-acetyl-l-cysteine (NAC) for 45 min followed by treatment with vehicle or 100 nM 5-HT for 5 min. Normalized data was analyzed for phospho-TrkB Y816. (D) Cells were incubated overnight with 0.01 or 0.1 µg/mL pertussis toxin (Ptx) followed by 5 min treatment with 0.1 µM 5-HT. (E) Cell cultures were pretreated with vehicle or 1 or 10 µM of the PDGF receptor kinase inhibitor AG 1296 for 45 min followed by treatment with vehicle or 100 nM 5-HT for 5 min. Western blots were evaluated for changes in phospho-TrkB Y816. (Data are representative of 5-6 independent experiments. * = p < 0.05 compared to vehicle-treated cells; # = p < 0.05 compared to 5-HT-treated cells, one-way ANOVA, Tukey post-test).

In addition to PDGF receptors, 5-HT receptors have been shown to trigger transactivation of fibroblast growth factor and epidermal growth factor receptors [33,34], but it is unknown if 5-HT can transactivate TrkB receptors, and whether ROS may be involved. Thus, we first determined whether TrkB phosphorylation is increased after H_2_O_2_ application. Indeed, similar to the PDGFβ receptor, TrkB phosphorylation at Y816 was increased in a dose-dependent manner with a maximum concentration of 0.1 µM H_2_O_2_ (Figure 4A). To determine if 5-HT could transactivate the TrkB receptor, we performed a time course of 5-HT application and, similar to the results with PDGFβ receptor transactivation, we observed maximum phosphorylation of the TrkB receptor after 5 min (Figure 4B). Given the similarity to PDGFβ receptor transactivation and the effect of H_2_O_2_ on TrkB receptor phosphorylation, we investigated whether 5-HT-induced TrkB receptor transactivation also required ROS. Indeed, pretreatment with *N*-acetyl-l-cysteine also blocked 5-HT-induced TrkB receptor transactivation (Figure 4C). Analogous to the 5-HT-PDGFβ receptor transactivation pathway [10], 0.1 µg/ml pertussis toxin also blocked 5-HT-induced TrkB receptor phosphorylation (Figure 4D), indicating a dependence on a Gα_i_-coupled 5-HT receptor. Although our previous data showed that the PDGF receptor kinase inhibitor AG 1296 blocked PDGFβ receptor transactivation by 5-HT [10], it did not block TrkB receptor transactivation (Figure 4E), suggesting that TrkB transactivation was not dependent on changes in PDGFβ receptor activity.

### The pathways for GPCR activation of ERK1/2 and RTK transactivation diverge at NADPH oxidase

**Figure 5:**
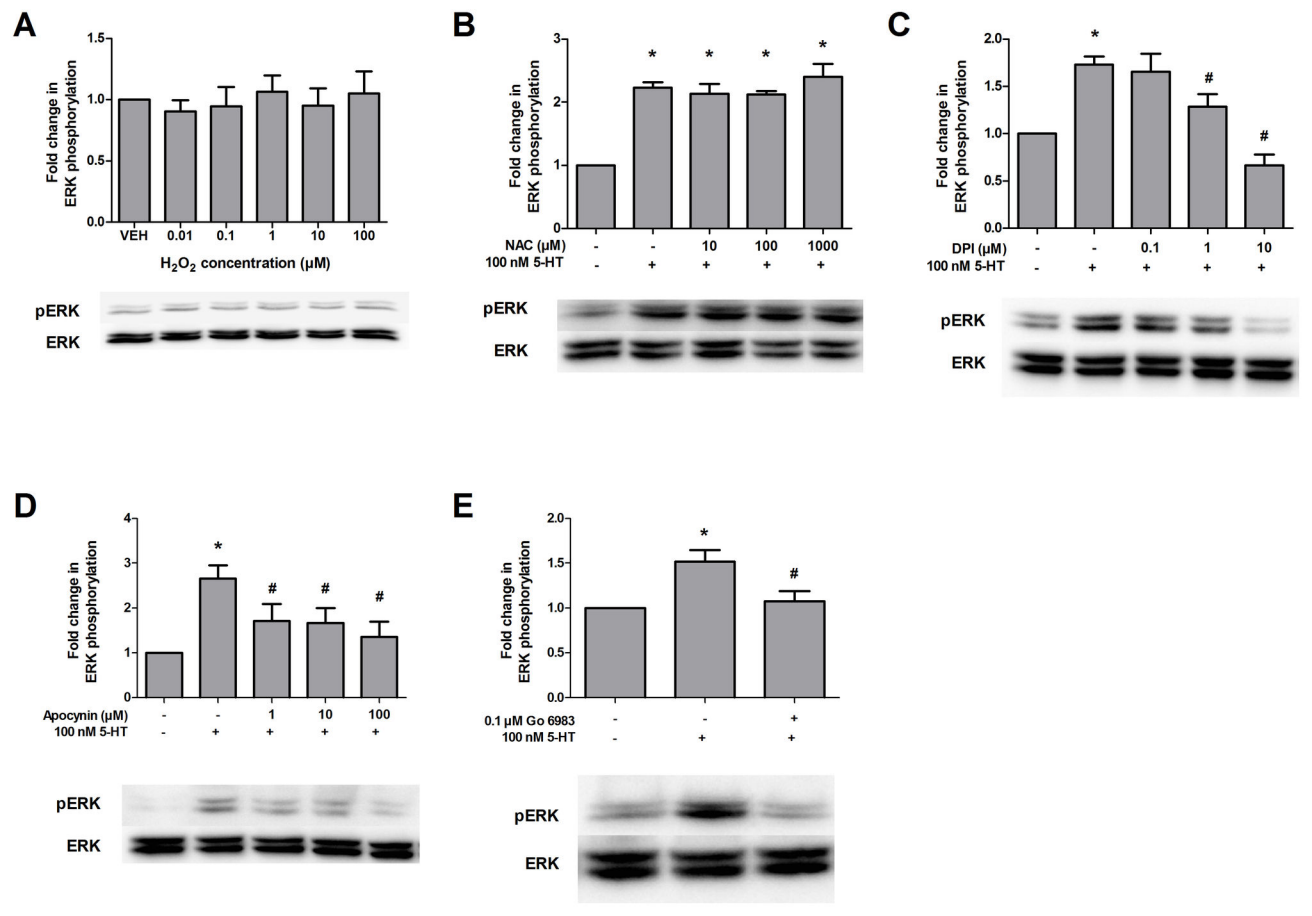
5-HT induced ERK1/2 phosphorylation diverges from the transactivation pathway at or after NADPH oxidase. (A) SH-SY5Y cells were treated with 0.01 to 100 µM H_2_O_2_ for 5 min. Following drug treatments, cell lysates were evaluated by Western blot analysis as described in Materials and Methods. Data were normalized to total ERK1/2 protein expression and are expressed as the fold change (average ± S.E.M.) in phospho-ERK immunoreactivity compared to vehicle-treated cells. (B) SH-SY5Y cell cultures were pretreated with vehicle or 10, 100 or 1000 µM of the ROS scavenger *N*-acetyl-l-cysteine (NAC) for 45 min followed by treatment with vehicle or 100 nM 5-HT for 5 min and lysates were evaluated as in “A”. Cell cultures were also pretreated with vehicle or the NADPH oxidase inhibitor diphenyleneiodonium chloride (DPI) (C) or apocynin (D) for 45 min followed by treatment with vehicle or 100 nM 5-HT for 5 min, and results were analyzed for phospho-ERK1/2 as described in “A”. (E) Cultures were pretreated with vehicle or 0.1 µM of the PKC inhibitor Go 6983 for 45 min followed by treatment with vehicle or 100 nM 5-HT for 5 min, and results were analyzed for phospho-ERK1/2 as described above. Representative blots of phospho-ERK1/2 and total ERK1/2 at 42 and 44 kDa are shown. (Data are representative of 4-8 independent experiments. * = p < 0.05 compared to vehicle-treated cells; # = p < 0.05 compared to 5-HT-treated cells, one-way ANOVA, Tukey post-test).

**Figure 6:**
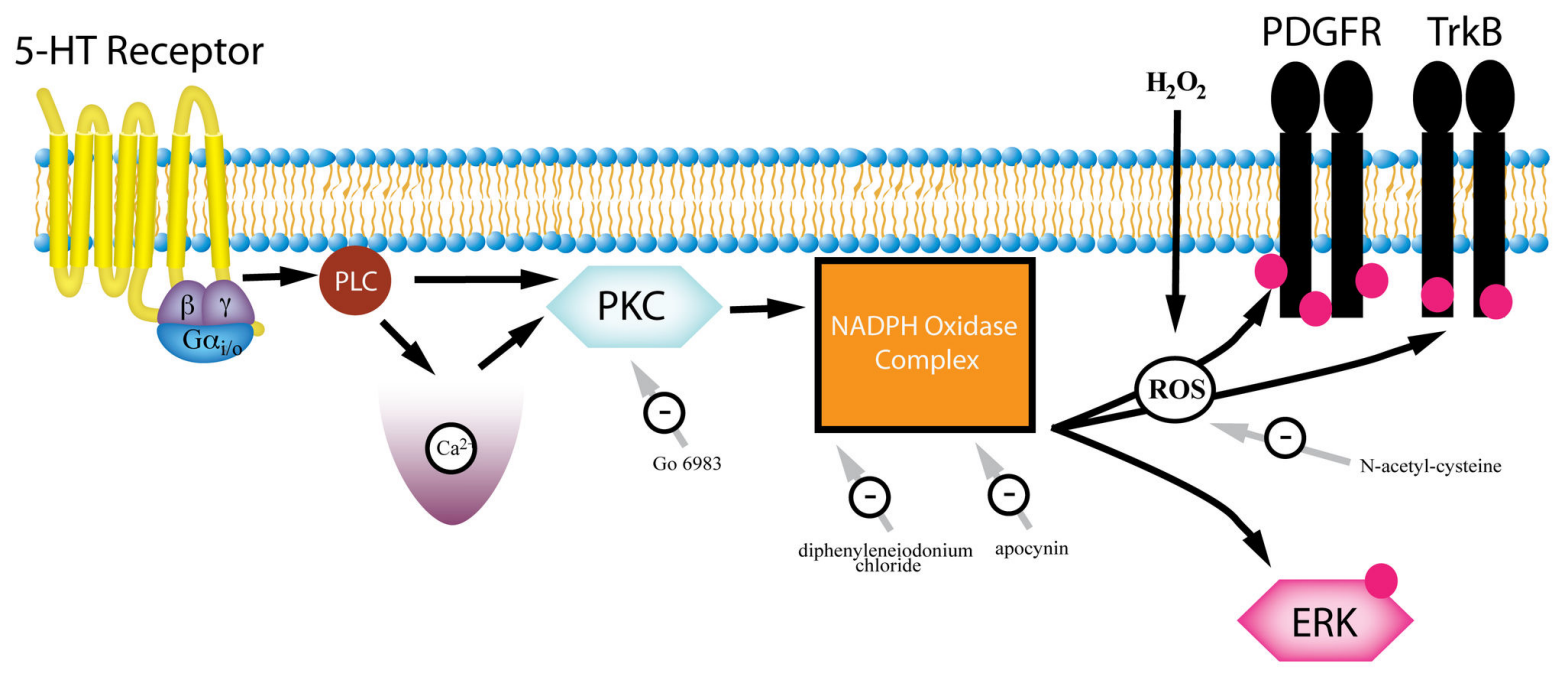
Mechanism of PDGFβ and TrkB receptor transactivation. Gα_i_-coupled GPCRs such as 5-HT_1A_ initiate transactivation signaling, which gets relayed through Gα or Gβγ subunits. PLC activation results in intracellular calcium release and activation of PKC. The NADPH oxidase subunits subsequently assemble and produce ROS. Active NADPH oxidase is required for both 5-HT-induced RTK and ERK1/2 phosphorylation but only endogenous ROS (or exogenous H_2_O_2_) is involved in RTK transactivation.

ERK1/2 is activated downstream of several RTKs and GPCRs, and RTK transactivation pathways have been proposed as a mechanism for GPCR to ERK signaling [12,22,23]. We have previously shown that the pathways for 5-HT-induced ERK1/2 phosphorylation and PDGFβ receptor transactivation are parallel: both involve Gα_i_, PLC, and intracellular calcium signaling [10]. However, these pathways must diverge at some point because PDGFβ receptor phosphorylation is not required for 5-HT-induced changes in ERK1/2 activity [10]. Given the results described above, we sought to determine whether 5-HT-induced ERK1/2 phosphorylation similarly involved ROS and NADPH oxidase. When SH-SY5Y cells were treated with H_2_O_2_, no significant increase in ERK1/2 phosphorylation was observed at any concentration tested (Figure 5A). H_2_O_2_ treatment also failed to induce ERK1/2 phosphorylation in primary cortical neurons (data not shown). Furthermore, in contrast to its ability to block 5-HT-induced PDGFβ and TrkB receptor phosphorylation, pretreatment with *N*-acetyl-l-cysteine had no effect on 5-HT-induced ERK1/2 phosphorylation (Figure 5B). However, the NADPH oxidase inhibitors, diphenyleneiodonium chloride and apocynin, as well as the PKC inhibitor Go 6983, blocked 5-HT-induced ERK1/2 phosphorylation (Figure 5C-E). This suggests that the divergence point for ERK1/2 phosphorylation and RTK transactivation occurs at or after NADPH oxidase, but upstream of ROS production (Figure 6).

## Discussion

The current report adds to a growing number of studies that have implicated ROS in the transactivation of RTKs [11,35,36]. There are several similarities in the pathways described for both 5-HT and ROS-induced increases in RTK phosphorylation. In both pathways, the phosphorylation of TrkB and PDGFβ receptors follows a similar dose response, and achieves a similar maximum fold change in phosphorylation compared to baseline. This, along with the ability of the ROS scavenger *N*-acetyl-l-cysteine to abrogate transactivation, suggests that ROS is a component of 5-HT-initiated transactivation pathways, and possibly other transactivation pathways as well. One of the striking differences between transactivation and direct ligand activation of the PDGFβ receptor is that the application of high concentrations of PDGF-BB can induce 10 to 100-fold increases in receptor phosphorylation [10] whereas for both 5-HT- and H_2_O_2_-mediated transactivation of PDGFβ receptor, the maximum observed increase in phosphorylation is only 1.5-2 fold.

Although we have identified ROS as being required for the transactivation of PDGFβ and TrkB receptors, the mechanism whereby ROS ultimately leads to increases in the phosphorylation state of the RTKs remains unknown. Some studies suggest that low levels of ROS act as second messengers capable of participating in intracellular signaling pathways [37,38]. ROS have the ability to oxidize catalytic cysteine residues in tyrosine phosphatase enzymes, such as the RTK phosphatase SHP-2, and the result of this oxidization is phosphatase inactivation [39,40]. These phosphatases possess a microenvironment that lowers the pK_a_ of the catalytic cysteine residue from the expected value of 8.5 to less than 5.5, sufficient for the thiol group to exist as a thiolate ion at physiological pH and to be sensitive to H_2_O_2_-induced oxidation [37]. This phosphatase inactivation is readily reversible and short-lived [39], which may explain why, if phosphatase inactivation is involved in RTK transactivation, the transactivation is transient [10]. Additional evidence supporting a role for SHP-2 in transactivation suggests that a knockdown of SHP-2 results in a greater basal phosphorylation of the epidermal growth factor receptor [39]. Since inhibition of PDGFβ receptor kinase activity in our system also abrogated 5-HT-induced PDGFβ receptor transactivation [10], we suspect that an increase in basal phosphorylation mediated by the receptor’s own kinase activity is responsible for the increase in phosphorylation observed, rather than through the action of a different kinase.

Since H_2_O_2_ has been implicated in the transactivation pathway of several RTKs, including PDGFβ and TrkB receptors shown here, it is conceivable that the physiological relevance of ROS in transactivation may ultimately consist of phosphorylating multiple RTKs via phosphatase inactivation, rather than specific single GPCR to single RTK pathways. If so, the *sum* of multiple small increases in RTK activation could lead to a greater increase in overall cellular RTK activity and the activation of their intracellular signaling pathways. The identification of ROS in transactivation pathways may also be an endogenous protective mechanism whereby an initial, mild cell stress and production of ROS protects the cell against subsequent more severe insults (and higher, toxic levels of ROS) by promoting the mitogenic effects of multiple RTKs. This is in line with other studies suggesting that transactivation is cytoprotective in the short term [41], whereas prolonged, chronic transactivation of growth factor receptors has been implicated in excessive mitogenic activity leading to disease states such as hypertension [42].

While the signaling steps downstream of ROS remain to be confirmed, we suggest that the upstream component responsible for ROS generation in transactivation pathways is NADPH oxidase. This enzyme is a large, multi-subunit complex that produces superoxide from oxygen and a donated electron from NADPH [30]. Superoxide dismutases then quickly convert superoxide to H_2_O_2_ [43]. Although often associated with respiratory burst in phagocytes [43], NADPH oxidase is active in non-phagocytic cells, with some subunits replaced with corresponding non-phagocytic homologs [30]. Among these subunits is Rac1, a member of the Rho GTPases family, which can be activated by both RTKs and GPCRs, and is required for oxidase activity [44,45]. Two studies have shown that PKC activates Rac1 [31,46], while other studies demonstrated that PKC can activate gp91 ^phox^/NOX2 (to enhance its association with other NADPH oxidase subunits) [47] and p47^phox^ [48]. Whether ROS formation by NADPH oxidase activity occurs intracellularly or extracellularly is still unclear in non-phagocytic cells, however some studies show NADPH oxidase assembles and functions in the cytoplasm, possibly in a vesicle or endoplasmic reticulum [49,50], which would result in intracellular ROS accumulation [51–53].

Our study failed to detect H_2_O_2_-induced increases in ERK1/2 phosphorylation, an observation that contradicts previous work showing that exogenously applied H_2_O_2_ results in ERK1/2 phosphorylation [54–56]. However, those reports used H_2_O_2_ concentrations between 0.1 and 2 mM – at least 100-fold higher than the concentrations used here. The low concentrations of H_2_O_2_ used in this study compared to other systems may not be sufficient to induce ERK1/2 phosphorylation, suggesting ROS is not required for ERK1/2 activation. This is further corroborated by the ROS scavenger *N*-acetyl-l-cysteine being able to block RTK phosphorylation, but not ERK1/2 phosphorylation, induced by 5-HT. Conversely, the NADPH oxidase inhibitors apocynin and diphenyleneiodonium chloride were able to inhibit ERK1/2 activation. These drugs may be preventing the assembly of the oxidase or chemically modifying the subunits [57,58], suggesting that the complete, functional oxidase is necessary for both PDGFβ receptor transactivation and ERK1/2 activation. Since the subunit Rac1 has been shown to activate MEK and subsequently ERK1/2 [31,59], it is conceivable that these drugs may be inhibiting the activity of subunits such as Rac1 and thus prevents both NADPH oxidase function and the phosphorylation and activation of ERK1/2.

We also show for the first time that 5-HT is capable of transactivating TrkB receptors. Like PDGFβ receptor transactivation [10], TrkB transactivation is sensitive to pertussis toxin, therefore it is dependent on Gα_i_-coupled 5-HT receptors. This is in line with other studies showing the dependency of transactivation on Gα_i_-linked GPCRs including D2-class dopamine [12], lysophosphatidic acid [15], and sphingosine-1-phosphate receptor-mediated transactivation [14], and may represent a general mechanism for transactivation initiation.

A diagram of the proposed signaling pathway is presented in Figure 6, which combines our data from previous work in the same systems [10]. Transactivation is initiated by Gα_i_-coupled GPCRs such as 5-HT_1A_ [10]. PLC is activated via the Gα_i_ and/or Gβγ subunits [60], which results in intracellular calcium release and activation of PKC. NADPH oxidase subunits assemble to produce ROS and the resulting H_2_O_2_ (or the exogenous application of H_2_O_2_) leads to RTK (PDGFβ and TrkB receptors) but not ERK1/2 phosphorylation. Crosstalk between 5-HT receptors and multiple RTKs may suggest that transactivation is a global pathway responsible for mitogenic or protective effects. In addition, the idea that serotonergic stimuli can activate neurotrophic factor and neuronal growth factor receptors brings together two major hypotheses for the pathophysiology of depression. Given that monoamine and neurotrophic hypotheses both propose a dysregulation in their respective signaling pathways as causes for clinical depression [21,61], it is possible that 5-HT-induced transactivation may improve symptoms by activating both serotonergic and neurotrophic signaling in the CNS.
